# Material and human resources for neonatal resuscitation in public maternity hospitals in Brazilian state capitals

**DOI:** 10.1590/S1516-31802008000300004

**Published:** 2008-05-01

**Authors:** Maria Fernanda Branco de Almeida, Ruth Guinsburg, José Orleans da Costa, Lêni Márcia Anchieta, Lincoln Marcelo Silveira Freire

**Keywords:** Infant, newborn, Cardiopulmonary resuscitation, Resuscitation, Perinatal care, Neonatal mortality, Recém-nascido, Ressuscitação cardiopulmonar, Ressuscitação, Assistência perinatal, Mortalidade neonatal

## Abstract

**CONTEXT AND OBJECTIVE:**

In 2002, the early neonatal mortality rate in Brazil was 12.42 per thousand live births. Perinatal asphyxia was the greatest cause of neonatal death (about 23%). This study aimed to evaluate the availability of the resources required for neonatal resuscitation in delivery rooms of public hospitals in Brazilian state capitals.

**DESIGN AND SETTING:**

Multicenter cross-sectional study involving 36 hospitals in 20 Brazilian state capitals in June 2003.

**METHODS:**

Each Brazilian region was represented by 1-4% of its live births. A local coordinator collected data regarding physical infrastructure, supplies and professionals available for neonatal resuscitation in the delivery room. The information was analyzed using the Statistical Package for the Social Sciences, version 10.

**RESULTS:**

Among the 36 hospitals, 89% were referral centers for high-risk pregnancies. Each institution had a monthly mean of 365 live births (3% < 1,500 g and 15% < 2,500 g). The 36 hospitals had 125 resuscitation tables (3-4 per hospital), all with overhead radiant heat, oxygen and vacuum sources. Appropriate equipment for pulmonary ventilation was available for more than 90% of the 125 resuscitation tables. On average, one pediatrician, three nurses and five nursing assistants per shift worked in the delivery rooms of each institution. Out of the 874 pediatricians and 1,037 nursing personnel that worked in the delivery rooms of the 36 hospitals, 94% and 22%, respectively, were trained in neonatal resuscitation.

**CONCLUSIONS:**

The main public maternity hospitals in Brazilian state capitals have the resources to resuscitate neonates at birth.

## RESUMO

**CONTEXTO E OBJETIVO:**

Em 2002, a mortalidade neonatal precoce brasileira foi 12,42 para cada mil nascidos vivos e a asfixia perinatal foi responsável por 23% dessas mortes. Este estudo visa avaliar a disponibilidade dos recursos necessários para a reanimação neonatal nas salas de parto de hospitais públicos brasileiros.

**TIPO DE ESTUDO E LOCAL:**

Estudo transversal multicêntrico de 36 maternidades, em 20 capitais brasileiras, em junho de 2003.

**MÉTODOS:**

As maternidades selecionadas em cada região brasileira representaram 1-4% dos nascidos vivos da região. O coordenador local da pesquisa respondeu a um questionário estruturado com dados a respeito da estrutura física, os equipamentos e o pessoal disponível para a reanimação neonatal em cada maternidade. A análise descritiva foi feita por meio do programa Statistical Package for Social Science 10.0.

**RESULTADOS:**

89% das 36 maternidades eram referência para gestação de risco. Cada hospital tinha um número médio mensal de 365 nascimentos (3% < 1.500 g e 15% < 2.500 g). Os 36 hospitais tinham 125 mesas de reanimação (3-4/hospital), todas com calor radiante, fonte de oxigênio e vácuo. Equipamento adequado para ventilação pulmonar estava disponível em mais de 90% das 125 mesas. Em média, um pediatra, três enfermeiras e cinco auxiliares de enfermagem trabalhavam por turno nas salas de parto de cada instituição. Dos 874 pediatras e 1.037 profissionais de enfermagem que atuavam nas salas de parto, 94% e 22% haviam recebido treinamento em reanimação neonatal respectivamente.

**CONCLUSÕES:**

As maternidades públicas das capitais brasileiras apresentam salas de parto com infra-estrutura adequada para a reanimação neonatal.

## INTRODUCTION

In 2002, there were 3,059,402 live births in Brazil, but 58,916 of these infants died before reaching one year of age.^1^ The early neonatal mortality rate was 12.42 deaths per thousand live births, and the late neonatal mortality rate was 3.62 deaths per thousand.^2^ Over the past decade, perinatal asphyxia was the greatest cause of death in the country, accounting for about 23% of the neonatal deaths.^3^

It has been estimated that the presence of adequate resuscitation procedures in delivery rooms may prevent the deaths of 359,000 newborns each year, around the world.^4^ Among the several established strategies for improving the care provided in delivery rooms, one of the most successful has been the joint proposal of the American Academy of Pediatrics and the American Heart Association, called the Neonatal Resuscitation Program (NRP).^5,6^ In Brazil, the Neonatal Resuscitation Program was started in 1994 by the Brazilian Pediatric Society (Sociedade Brasileira de Pediatria, SBP) and, by the end of 2003, 388 instructors had already taught 1,123 theoretical-practical training courses throughout the country, to about 24,000 healthcare professionals acting in delivery rooms.^7^

## OBJECTIVE

To investigate the availability of physical, material and human resources for resuscitating newborn infants in the delivery rooms of public maternity hospitals in Brazilian state capitals.

## METHODS

### Type of study

This was a cross-sectional study, with prospective collection of data from June 1 to 30, 2003. The project was approved by the Research Ethics Committees of the institutions with which the principal investigators were associated, and by the Clinical Board of Directors of each participating institution.

### Settings and sample

Since 97% of Brazilian births occur in hospitals,^1^ public maternity hospitals in the state capitals were selected according to the following criteria: state capitals with more than 5,000 live births in 2002;^1^ public maternity hospitals with the highest numbers of births in each capital. Thus, public maternity hospitals with the highest numbers of births per month in the capitals of the northern, northeastern and central-western regions were invited to participate in the study. In the southeastern and southern regions, public maternity hospitals with the highest number of births in the main geographical areas of the cities were invited to participate. The number of live births per month in these maternity hospitals was intended to represent 4-8% of the births in each Brazilian region. Thirty-six maternity hospitals in 20 Brazilian state capitals were invited and agreed to participate in the study (Table 1).

**Table 1.** Distribution of the 36 Brazilian maternity hospitals included in the study, according to the geographical location and estimated number of live births per month^1^

Region	Live births per month per region in 2002	State Capitals	Number of maternity hospitals included	Estimated live births per month
**North**	25,101	Manaus	1	500
		Belém	2	480
**Northeast**	77,476	São Luís	2	1,000
		Teresina	1	900
		Fortaleza	3	1,600
		Natal	1	500
		Recife	1	300
		Maceió	1	300
		Aracajú	1	200
		Salvador	1	600
**Southeast**	99,597	Belo Horizonte	2	750
		Vitória	1	350
		Rio de Janeiro	4	1,650
		São Paulo	7	2,250
**South**	33,843	Curitiba	2	900
		Florianópolis	1	400
		Porto Alegre	2	1,350
**Center-west**	18,933	Campo Grande	1	350
		Goiânia	1	200
		Brasília	1	450

### Procedures and main measurements

Written questionnaires asking about the structure of each hospital were answered by a site research coordinator. The accuracy of the responses was checked by each state coordinator within the Brazilian Neonatal Resuscitation Program. At each maternity hospital, the following data were collected: institutional characteristics, characterization of the neonatal patients under care, number of delivery rooms, number and characteristics of the delivery tables for neonatal resuscitation, availability of transport incubators, supplies available for each resuscitation table (in accordance with the recommendations from the Ministry of Health in 1994^8^ and 2001^9^) and human resources for neonatal care in the delivery room.

### Statistical methods

After checking the consistency of data entry from the worksheets, descriptive analysis of the physical, material and human resources that exist was performed using the Statistical Package for the Social Sciences (SPSS), version 10.0.

## RESULTS

Out of the 36 institutions, 19 (53%) were general hospitals and 17 (47%) were maternity hospitals; 24 (67%) were participating in the “Baby-Friendly Hospital Initiative” and 32 (89%) were referral centers for high-risk pregnancies. More than 90% of the patients cared for in these institutions had Brazilian public health insurance. In addition, 23 (64%) of the institutions were teaching sites for undergraduate students in medicine and nursing, 27 (75%) for residents in pediatrics and 10 (28%) for neonatal fellows.

The number of beds allotted to newborns in the 36 maternity hospitals, described as medians (with minimums and maximums), was 52 (0-117) for rooming-in, 18 (3-54) for intermediate care, 12 (0-28) for intensive care, and four (0-20) for kangaroo care. The mean number of neonates cared for in each institution per month from January to June 2003 was 365 (95% confidence interval, CI: 285-446), among which 15% were low birth weight newborn infants (< 2,500 g) and 3% were very low birth weight (< 1,500 g).

Each maternity hospital had an average of four delivery rooms (95% CI: 4-5) and three neonatal resuscitation tables (95% CI: 3-4). In total, the 36 institutions had 125 resuscitation tables. On average, the area available for each resuscitation table was 4.2 m^2^ (95% CI: 3.7-4.7). The distance between the resuscitation table and the birthing bed was 2.7 m (95% CI: 2.3-3.2), and the distance from the resuscitation table to the neonatal intensive care unit (NICU) was 41 m (95% CI: 35-48). A transport incubator with oxygen tank was reported to be available in the obstetric center of 29 maternity hospitals (81%).

Out of the 125 resuscitation tables, 82 (66%) were of an appropriate height for the pediatrician to perform ventilation and chest compressions, and 93 (74%) had three sides to access the newborn. All tables had an overhead source of radiant heat, a source of oxygen (99% with a flow meter, 95% with unheated humidification and 2% with humidification and heating) and a source of vacuum (82% with a manometer).

In relation to airway aspiration supplies, 122 (98%) of the tables had suction catheters, 30 (24%) used bulbs and 73 (60%) had a meconium aspiration device. Regarding the supplies for providing free-flow oxygen, on 108 (86%) of the tables this was made available by latex oxygen tubing, on 36 (29%) by facial masks and on 24 (19%) by flow-inflating bags. A self-inflating bag with a volume of 250 to 750 ml was present in 123 tables (93%). Facial masks for full-term infants were available on 120 (96%) resuscitation tables and for preterm infants on 113 (90%). Inappropriate facial masks for resuscitation of term and preterm neonates (regarding format, material and/or size) were observed on 23 (19%) and 34 (30%) of the tables, respectively.

In terms of supplies for tracheal intubation, 116 (93%) of the resuscitation tables had a laryngoscope, 115 (92%) had 2.5 mm tubes, 118 (94%) had 3.0 and 3.5 mm tubes and 96 (77%) had 4.0 mm tubes. All of them were disposable and had a uniform diameter.

The main medications for neonatal resuscitation in the delivery rooms were available for prompt use in all institutions: out of the 125 resuscitation tables, 110 (88%) had 1/1,000 adrenaline vials, 68 (54%) had diluted adrenaline (1/10,000), 116 (93%) had normal saline, 107 (86%) had sodium bicarbonate and 93 (74%) had naloxone. Of the 125 tables, 89 (71%) had supplies for umbilical catheterization, and specific umbilical catheters were available on 33 of them.

The number of professionals participating in newborn care provision in the delivery room, described as means (with minimums and maximums) per institution, were: one pediatricians per shift (1-2), 3.5 nurses during day shifts (0-14), two nurses during night shifts (0-17), six nursing technicians during day shifts (0-44) and 4.5 nursing technicians during night shifts (0-44). The pediatric teams covered day and night shifts during the week and weekends. A total of 920 pediatricians and 1,187 nursing personnel worked at the 36 institutions and, respectively, 874 (95%) and 1,037 (87%) answered the questionnaire regarding their neonatal resuscitation training. Of these, 821 (94%) pediatricians had completed at least one neonatal resuscitation training course in accordance with Brazilian Pediatric Society guidelines and 405 (54%) of them had had their most recent training course within the two years preceding this study. Only 228 (22%) of the 1,037 nursing personnel had had at least one neonatal resuscitation training course. These results are shown in Table 2.

**Table 2.** Characteristics of the human resources in charge of newborn care in the delivery rooms of the 36 Brazilian maternity hospitals

	Pediatricians n = 874	Nurses n = 179	Technicians n = 811	Midwives n = 47
Age (years)	40 ± 8	36 ± 8	40 ± 9	38 ± 9
Female	77%	91%	95%	98%
Experience in delivery room (years)	12 ± 8	6 ± 5	7 ± 7	11 ± 7
Resuscitation training*	94%	50%	14%	64%

* At least one eight-hour theoretical and practical neonatal resuscitation training course given by Brazilian Neonatal Resuscitation Program instructors.

## DISCUSSION

According to several studies,^5,10–13^ about 10-20% of live births need positive pressure ventilation in the delivery room. Of these neonates, 90% improve only with bag and mask ventilation and the other 10% need tracheal intubation. About 1% of resuscitated neonates or 0.3% of all live births not only need ventilation but also need chest compressions and/or medication in the delivery room. Considering that 97% of the deliveries in Brazil in 2002 occurred in hospitals^1^ and that, as described above, there is a frequent need for some neonatal resuscitation procedure, it is important for the institutions in which these children are born to be equipped with the physical, material and human resources to enable resuscitation.

Out of the 36 institutions, almost 90% were teaching hospitals and their settings form models for physicians and nurses in training.^14^ An average of 300 to 400 births per month occurred in each maternity hospital: 15% of the newborns had low birth weight and 3% had very low birth weight. These percentages confirm that these institutions were referral centers for high-risk pregnancies, since the overall rates for low and very low birth weight in Brazil were 8% and 1%, respectively, in 2003.^1^

The average area available for each resuscitation table in each institution (4.2 m^2^) was smaller than the 6 m^2^ prescribed by the Ministry of Health.^15^ The distance between the neonatal resuscitation table and the birthing bed was seen to be appropriate (less than 3 m). However, the reported distance of 41 m between the resuscitation units and the NICU indicates the need for an equipped transport incubator for safe intra-hospital transportation of the newborn. In fact, 80% of the maternity hospitals had one transport incubator with oxygen in their obstetric centers.

In relation to the resuscitation units themselves, all of them had a source of radiant heat. However, 40% of them presented a height lower than 90 cm: below this height, the tracheal intubation procedure becomes difficult. In addition, 26% of them only had access to the patient on one or two sides, thus making intensive resuscitation interventions troublesome.^16^

A source of oxygen was present in all units, of which 95% were humidified and 2% were also heated. The low supply of heated oxygen humidifiers on the Brazilian market is a result of their high cost as well as their low durability, and these factors are obstacles to better neonatal care in the delivery room. Despite these points, all the 125 neonatal resuscitation tables had at least the basic requirements for properly resuscitating newborns: overhead radiant heat, oxygen and vacuum.

In view of the recommendation from the Brazilian Neonatal Resuscitation Program that the appropriate type of catheter for airway aspiration is the suction catheter, the finding that tubes of this type were present in all units shows that this teaching has been incorporated into clinical practice. On the other hand, the fact that 60% of the resuscitation units had a meconium aspiration device reflects the difficulty of incorporating the teachings when the industry's supply of materials does not follow the established recommendations.^8^

Ventilation is the most effective procedure in neonatal resuscitation in the delivery room. The vast majority of the 125 resuscitation tables had appropriate self-inflating bags with some safety feature. As previously mentioned, only about 1% of resuscitated neonates or 0.3% of live births need chest compressions and/or medication in the delivery room in addition to ventilation, when cared for by qualified personnel.^5,10^ These 36 hospitals had at least one neonatal resuscitation table with all the equipment needed to perform advanced resuscitation procedures.

So far, the picture that has been taken of these 36 public maternity hospitals accounting for 4-8% of the live births in each Brazilian region shows that the basic physical and material resources for neonatal resuscitation were available not only in most institutions, but also on most resuscitation tables in each institution. Still, to use these resources, maternity hospitals also need to have a team of healthcare professionals caring for mothers and their newborns on a daily basis.

The Regional Board of Medicine of the State of São Paulo found in 1997 that 70% of the 99 hospitals in the State did not have a pediatrician to assist newborns in delivery rooms.^17^ In one region of the State of São Paulo in 2001, 82% of the 11 maternity hospitals in the region only had pediatricians to attend newborn delivery rooms “at a distance”.^18^ In the present study, it was not only found that pediatricians were present in the delivery room, but also that 94% of them had had at least one neonatal resuscitation training course. On the other hand, the nursing staff was not equally trained. This lack of training may impair effective resuscitation of the newly born infant.

Although the picture taken at these maternity hospitals is optimistic regarding the availability of material and medical resources to care for newly born infants, it should be stressed that these results were obtained mainly in tertiary teaching hospitals. It is important to follow this investigation with other studies of maternity hospitals that are not referral facilities for high-risk pregnancies.

## CONCLUSION

The main public maternity hospitals in the Brazilian state capitals in 2003 had sufficient physical, material and human resources for neonatal resuscitation in their delivery rooms. Efforts should be directed towards training the nursing staff in order to have a team of professionals with the skills to effectively resuscitate newly born infants.
